# Supplementary material for: Revealing the Organ-Specific Expression of SPTBN1 using Single-Cell RNA Sequencing Analysis
Source: bioRxiv. 2023 Jun 5:2023.06.01.543198. Preprint. [Version 1] doi: 10.1101/2023.06.01.543198 (PMC10274633; doi:10.1101/2023.06.01.543198)
Supplement: Supplement 1 [file NIHPP2023.06.01.543198v1-supplement-1.pdf]

Supplemental Figure

Figure S1. Violin plot for the quality control in each organ type.

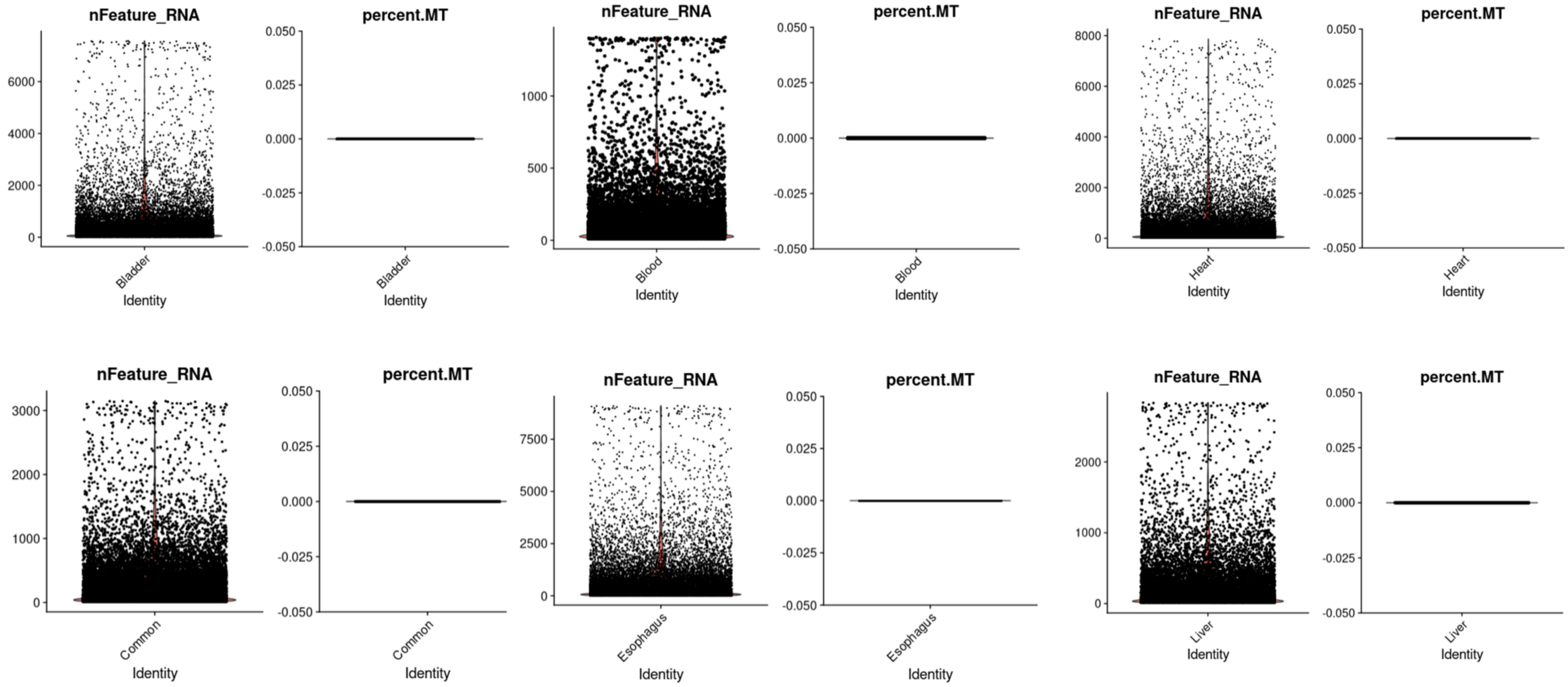

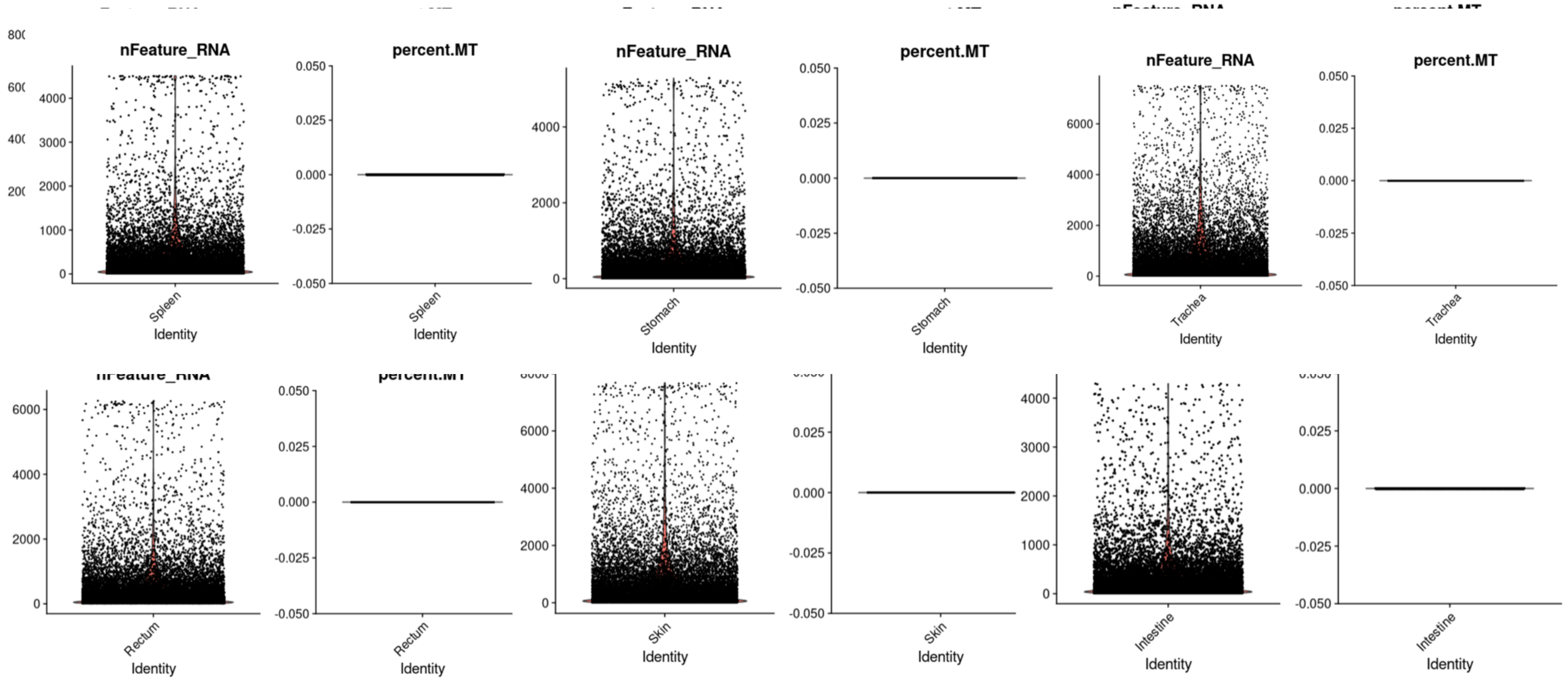
nFeature_RNA
percent.MT
Spleen
Identity
nFeature_RNA
percent.MT
Stomach
Identity
nFeature_RNA
percent.MT
Trachea
Identity
percent.MT
Rectum
Identity
Skin
Identity
Intestine
Identity

Figure S2. Violin plots of the normalized expression of *PKDCC* for each organ by the cell type.

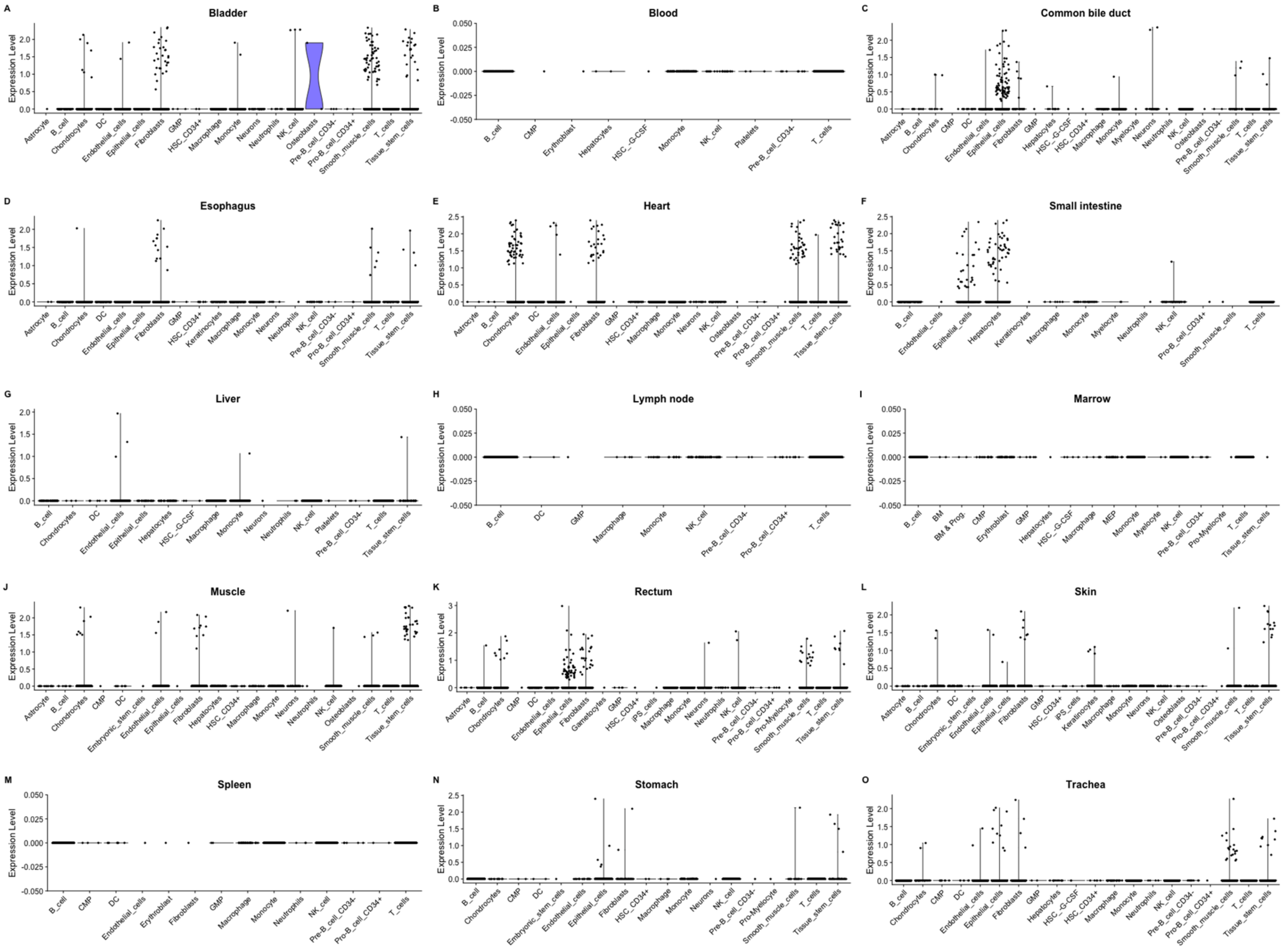
A
Bladder
B
Blood
C
Common bile duct
D
Esophagus
E
Heart
F
Small intestine
G
Liver
H
Lymph node
I
Marrow
J
Muscle
K
Rectum
L
Skin
M
Spleen
N
Stomach
O
Trachea
Expression Level

Figure S3. Violin plots of the normalized expression of *EPDR1* for each organ by the cell type.

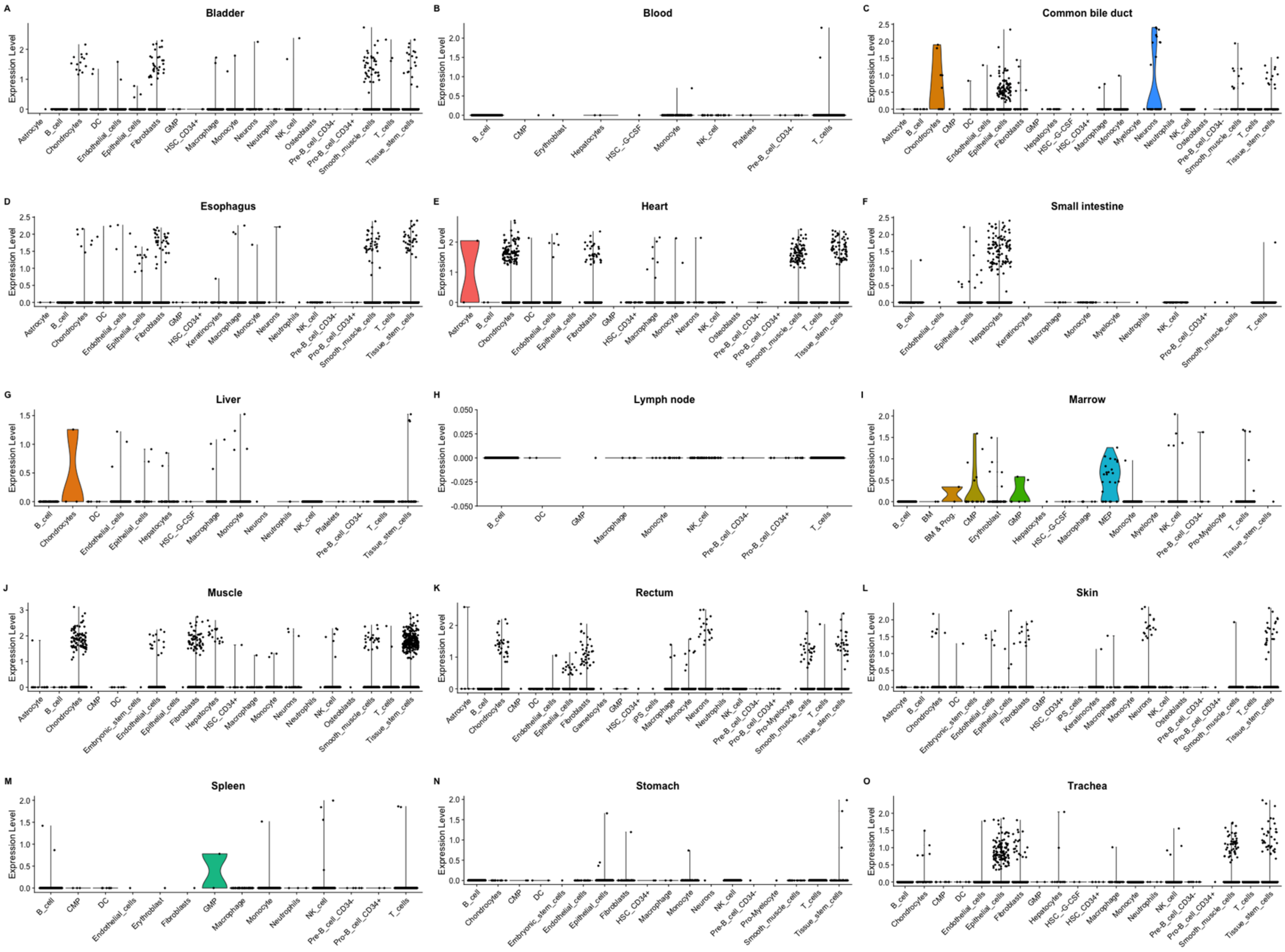
A
Bladder
B
Blood
C
Common bile duct
D
Esophagus
E
Heart
F
Small intestine
G
Liver
H
Lymph node
I
Marrow
J
Muscle
K
Rectum
L
Skin
M
Spleen
N
Stomach
O
Trachea
Expression Level
